# Analytic and Diagnostic Performances of Human Papillomavirus *E6/E7* mRNA Test on up-to 11-Year-Old Liquid-Based Cervical Samples. A Biobank-Based Longitudinal Study

**DOI:** 10.3390/ijms18071480

**Published:** 2017-07-11

**Authors:** Roberta Zappacosta, Francesca Sablone, Lucia Pansa, Davide Buca, Danilo Buca, Sandra Rosini

**Affiliations:** 1 Department of Medical, Oral Sciences and Biotechnology, G d’Annunzio University of Chieti-Pescara, Via dei Vestini, 66100 Chieti, Italy; luciapansa@libero.it (L.P.); bucadavide@gmail.com (D.B.); rosini@unich.it (S.R.); 2 Surgical Pathology Unit, SS Annunziata Hospital, Via dei Vestini, 66100 Chieti, Italy; francescasablone@hotmail.it; 3 School of Clinical Biochemistry, G. d’Annunzio University of Chieti-Pescara, Via dei Vestini, 66100 Chieti, Italy; 4 Department of Medicine and Aging Sciences, G d’Annunzio University of Chieti-Pescara, Via dei Vestini, 66100 Chieti, Italy; danilooh@hotmail.com

**Keywords:** cervical cytology biobank, RNA stability, *E6/E7* mRNA testing, retrospective longitudinal study, triage test, personalised medicine

## Abstract

Human Papillomavirus (HPV) *E6/E7* mRNA test demonstrated high specificity in detecting HPV infections, but studies assessing its efficacy in terms of cancer risk stratification are lacking. Follow-up studies are arduous and expensive. Biobank would be the answer to the problem, although data investigating the effects of long-term storage on RNA preservation are still needed. We addressed these issues by retrieving 202 residual liquid-based cervical specimens, collected from 149 women attending cervical cancer screening during the years 2001–2012. Samples were stored in Adriatic Biobank at room temperature and without any handing. After calculation of RNA yield and purity, *E6/E7* mRNA test was retrospectively performed on each samples, to assess analytic and diagnostic performances. Using automated extraction procedures, RNA of good quantity and quality was obtained. The mean value of RNA concentration was 27.5 ng/μL. The mean A260/A280 ratio was 2.1. An invalid mRNA test result was found in 11.9% of the specimens. Neither RNA integrity, nor analytic performances of mRNA test were influenced by the year of sample collection. In total, 62.4% of the specimens tested as mRNA positive; among these, 89.2% were CIN2+. *E6/E7* mRNA was detected in all Squamous Cervical Cancer (SCC) cases. Percentage of positive samples increased with the severity of histological diagnosis. mRNA testing, showing specificity and predictive values of 75.6% and 84.4%, respectively, significantly improved the corresponding values for DNA testing. Thus, the reflex mRNA test was demonstrated to be suitable to triage women with persistent cervical lesions. A “one sample for all” approach is possible, with practical benefits for Biobank-based long-term longitudinal studies, diseases prevention, prediction, diagnosis and treatment.

## 1. Introduction

Infection with oncogenic Human Papillomavirus (HPV) has been recognised as the leading cause of cervical intraepithelial lesions (CIN) and invasive cancer [1], even if only a minority of infections ever results in squamous cell carcinoma (SCC) [2,3].

Viral infection is a necessary but far from sufficient cause during carcinogenesis.

The introduction of HPV-DNA test in the diagnostic algorithm of patients with cervical lesions improved the effectiveness of cervical cancer screening. In randomized controlled trials DNA testing demonstrated high sensitivity in detecting CINs, thus reducing both cervical cancer (CC) incidence and mortality [4,5]. As a counterpart, the main problem with HPV-DNA test remains the high prevalence of HPV infection in comparison with the low number of women developing cancer. The low specificity of HPV-DNA test does not allow us to distinguish between transient infections, which usually clear within two years, and persistent infections, which harbour a potential highest risk of progression toward CC [6]. The current concept of CC prevention aims to identify not just the mere HPV infections but, among these, clinically relevant lesions, which would benefit from medical interventions.

It is therefore important to focus on a supplementary test, able to limit follow-up and over-treatments, at the same time giving additional information about the outcome of the disease [3,7].

A key factor inducing CC is the integration of HPV genome into the human genome because of persistent infections [8]. Viral persistence, often caused by loss of immune control, is essential for HPV-mediated cancerogenesis. Persistence would act through disruption of cell-cycle control and accumulation of genetic aberrations [9]. In transforming CINs, the HPV cycle is aborted, but *E6* and *E7* early genes are overexpressed in proliferating cells. *E6* and *E7* genes encode for proteins, which are able to modify the expression of cell cycle controllers and DNA repair regulators [10,11,12,13]. They act as oncoproteins.

The carcinogenic role of *E6* and *E7* oncoproteins was confirmed by numerous studies demonstrating their implication in initiation and maintenance of malignant phenotype [14,15,16]. The rationale for detecting *E6/E7* transcripts from oncogenic HPV types is to reduce the prevalence of clinically irrelevant lesions (thus implementing the specificity of cervical cancer screening), at the same time increasing the positive predictive value of DNA testing [17]. In summary, an *E6/E7*-based test should be able to stratify the risk [18].

Before integrating a molecular biomarker into clinical practice, a five-phase framework has been proposed [7,19,20]. At present, most biomarkers are in phase 1 and 2. Phase 3 consists of retrospective longitudinal repository studies. A series of multiple biospecimens would offer an interesting support to address pending questions on the use of predictive tools. Biobanks, lying in the systematic storage of human specimens and associated data, presently represent a key platform for personalized medicine; they would support scientific progress in risk stratification, biomarker discovery and tests validation. In particular, the Cervical Cytology Biobank (CCB) would be useful for large studies evaluating prognostic markers involved in cervical carcinogenesis [21,22].

Molecular analyses on cytological samples is currently feasible because of the introduction of Liquid-based Cytology (LBC) [22]; on the other hand, knowledge is lacking regarding: (i) sample stability of archived specimens beyond three years from baseline time of collection; (ii) suitability of old residual LBC samples for mRNA testing [23]. These data are strongly required.

The goals of this retrospective longitudinal study were to assess the analytic performances of Nuclisens EasyQ HPV *E6/E7* mRNA test on up to 11-year-old residual PreservCyt cervical samples, and to estimate its clinical accuracy in predicting CIN2+ lesions.

## 2. Results

**Figure 1:**
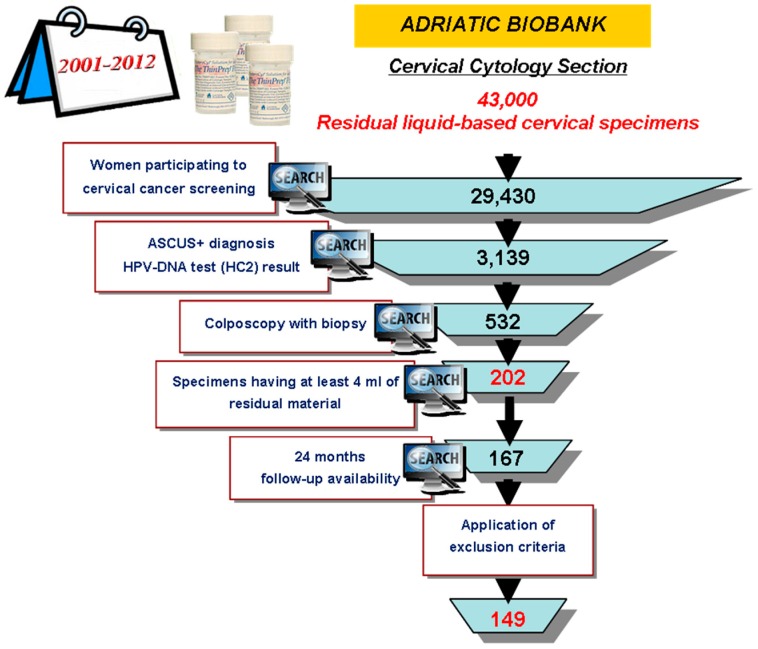
Cases recruitment funnel. RED text indicates the most relevant data for the study population.

Figure 1 shows a detailed description of the whole study population.

The analytic performances of the mRNA test were evaluated on 202 residual cervical specimens.

The mean time from LBC collection of LBC was 66.7 ± 35.2 SD months (median, 60 months; range 1–132). Among LBC specimens, the mean value of RNA recovery was 27.5 ng/μL and the mean A260/A280 ratio was 2.1. Fresh-frozen tissue showed RNA concentration of 19.4 ng/μL; A260/280 ratio was 1.8. The Snap-frozen tissues revealed mean values for RNA yield and purity of 72 ng/μL, and 2.0, respectively. Finally, RNA yields for LBC specimens stored at 4 and −20 °C were 28.6 ng/μL and 22.7 ng/μL, respectively, with a purity value of 2.09 and 2.1, respectively.

**Figure 2:**
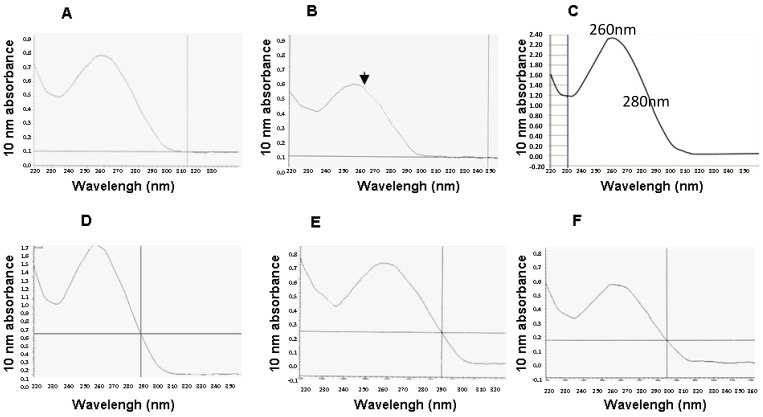
Comparison of spectrophotometric RNA profiles. (**A**) one of the Liquid-based Cytology (LBC) samples collected in 2002 and stored at RT; (**B**) fresh-frozen tissue specimen. Black arrow identifies a budge on the peak shoulder; (**C**) an ideal nucleic RNA sample, as described by Nanodrop manufacturer’s manual [24]; (**D**) one of the six snap-frozen tissue specimens; (**E**) residual LBC specimen stored at 4 °C; (**F**) residual LBC sample stored at −20 °C.

Figure 2 compares spectral RNA patterns obtained by samples with different methods of preservation. Curve C represents the absorbance spectrum of a highly pure RNA sample with close to ideal A260/280 ratio (2.07), as described in Nanodrop manufacture’s manual [24].

Curve B shows the spectrophotometric RNA profile obtained by the fresh-frozen sample. The budge of the peak shoulder at 260 nm was most likely due to contamination.

Analysis of results from mRNA testing showed amplification of *U1A* gene in 88.1% (178/202) of the specimens. In 11.9% (24/202) of the cases, U1A was not detected; consequently, the mRNA test result was encoded as undetermined/invalid. Among this group, the mean value for RNA concentration was 4.3 ng/μ, and HPV *E6/E7* mRNA has been found in 37.5% (9/24) of the specimens. No SCC was associated with an undetermined mRNA test result.

U1A mRNA was detected in 100% (6/6) of the snap-frozen specimens, in fresh-frozen tissue, as well as in specimens stored at 4 and −20 °C, respectively.

**Figure 3:**
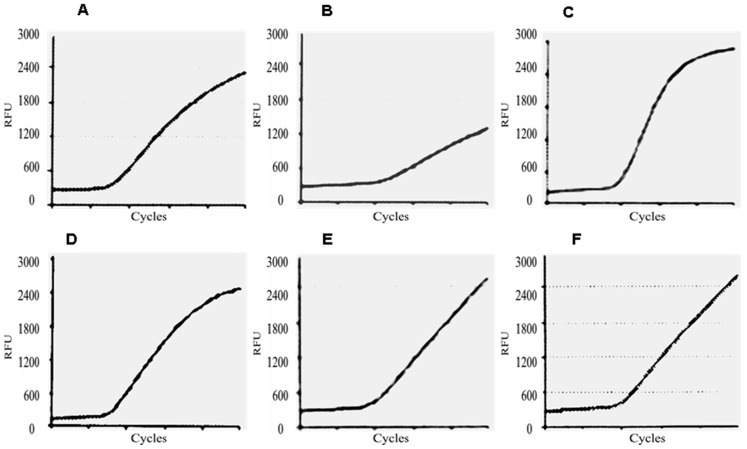
Real-time (RT) kinetic curves related to *U1A* gene from the different samples included in the study. (**A**) one of the residual LBC specimen collected in 2002 and stored at RT; (**B**) fresh-frozen tissue; (**C**) positive control included in the kit; (**D**) one of the six snap-frozen tissues; (**E**) residual LBC specimen stored at 4 °C; (**F**) residual LBC sample stored at −20 °C. *U1A* was detected by Texas Red (TxR) fluorophore. RFU, Relative Fluorescence Unit.

Figure 3 shows the comparison of profiles from *U1A* amplification obtained by samples having different storage conditions. Curve C is related to one of the five positive controls included in the kit, consisting of artificial *E6/E7*-target sequences specific for HPV-16. The curve related to the fresh-frozen specimen (B) demonstrated amplification occurring later than expected. This would most likely be due to the low concentration and quality of purified RNA.

**Figure 4:**
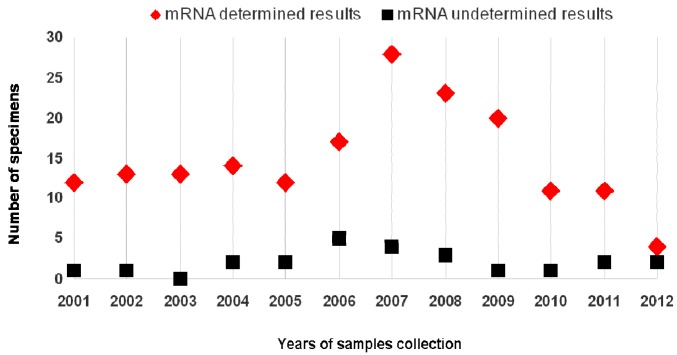
Results from mRNA testing in relation with the year of specimens’ collection.

Figure 4 shows the distribution of mRNA test results, dichotomized as determined/undetermined, in relation with the years of LBC samples collection.

Although 79.2% of undetermined results dropped in samples collected between 2001 and 2009, a week correlation has been found between the time of specimen collection and the integrity of RNA (as verified by *U1A* gene amplification) (*r* = −0.06). To address the trend, we clustered the years of collections by three periods (2001–2004, 2005–2008, 2009–2012) (Table 1). No statistical significance was found (*p* = 0.38).

In order to provide comparative data, we also analysed a series of 202 consecutive cervical samples undergoing mRNA test within six weeks from collection, accordingly with manufacturer’s recommendations. These specimens were related to patients harbouring ASCUS+ lesions (mean age, 39 ± 11.8 SD years; range 21–79 years; median 37 years). The proportion of women <30 years was 24.3% (*n* = 49/202). The prevalence of oncogenic HPV-DNA positivity was 75.7% (*n* = 153/202). 

Qualifying the test as determined when *E6/E7* mRNA from one or more HPVs were detected, even in absence of U1A amplification, the rate of undetermined results was 4.95%. Conversely, considering as determined only tests showing U1A amplification, the percentage of undetermined results increased to 13.3%. Among determined test results, mRNA positivity has been found in 50% of the samples collection (*n* = 88/175) and in 54.9% (*n* = 84/153) of the HPV-DNA positive cases.

Diagnostic performances of *E6/E7* mRNA test were evaluated on a subset of 149 patients (Figure 1). Patients ranged from 20 to 67 years (mean, 36.7 ± 10.7 SD years; median, 34 years). 30.9% (*n* = 46) of women were below 30 years of age, 69.1% *n* = 103) aged 30 or above.

Considering cytological diagnosis, 44 ASCUS (29.5%), 53 LSIL (35.6%), 43 HSIL (28.9%) and 9 SCC (6%) were detected. Histological assessment showed 56 (37.6%) CIN2− and 93 (62.4%) CIN2+. Among the latest group, 32 CIN2, 52 CIN3 and 9 SCC were included.

A summary of the distribution of patients according to cytological diagnosis, histological diagnosis, HPV-DNA and *E6/E7* mRNA testing is represented in Table 2.

Ninety-three (62.4%) specimens tested as mRNA positive; among these, eighty-three (89.2%) showed CIN2+. *E6/E7* mRNA was detected in all the SCC cases.

mRNA negativity has been found in 32 (21.6%) women; among these, 31 (96.9%) showed CIN2−. Percentage of positive samples increased with the severity of histological diagnosis (*p* < 0.01). HPV-16 resulted as the most prevalent genotype (64.5%); 100% of SCC cases tested as HPV-16 positive. In nine cases, more than one *E6/E7* mRNA transcripts were detected; among these, eight patients (88.9%) were CIN2+.

mRNA test result was associated with CIN2+ with an Odds Ratio (OR) risk of 257.3 (95% CI: 31.6-2094). All of the CIN2−/mRNA positive cases (10.8%) persisted over time. The only CIN2+/mRNA negative case that cleared over time was related to a patient younger than 30 at the time of histological diagnosis. To investigate the effect of age on the clinical performances of mRNA testing, sensitivity, specificity, positive predictive value (PPV) and negative predictive value (NPV) were calculated independently for women below 30 years of age, and women aged 30 years or older. Women who had an undetermined mRNA result were excluded from this calculation. Among patients below 30, 29% (*n* = 11) had CIN2−, and 71% (*n* = 27) had CIN2+. Among patients aging 30 or above, 34.5% (*n* = 30) were CIN2−, and 65.5% (*n* = 57) were CIN2+ (*p* = 0.4). All of SCC occurred in the oldest patients. Specificity and PPV of the mRNA test was higher in women aged 30 or above (Table 3), but difference did not reach statistical significance (*p* = 0.97).

Considering the whole study population, DNA positivity has been found in 90.6% (*n* = 135/149) of the patients; among these, 67.4% were CIN2+. Sensitivity, specificity, PPV and NPV with 95% CIs were 98% (91.7–99.6), 21%, 4% (12–34.8), 67% (56.7–75) and 86% (56.2–97.5), respectively.

Excluding mRNA undetermined results from the calculation, HPV-DNA positivity has been found in 92.8% (116/125) of the samples; among these, 70.7% (82/116) were CIN2+. All of the SCCs showed DNA positive result. DNA negativity was detected in 7.2% (9/125) of the specimens and in 17% (7/41) of CIN2−. The exclusion of undetermined mRNA cases did not significantly inflate the sensitivity of DNA testing (*p* = 0.8).

A positive DNA test result conferred a CIN2+ OR risk of 8.44 (95% CI: 1.7–42.7). In total, 78.4% (91/116) of patients showing DNA positive results tested as mRNA positive. Two samples were mRNA positive/DNA negative. Both cases were related to women below 30, bearing a CIN2+, and infected by HPV-16.

Percent agreement between DNA and mRNA test was 78.4% (*k* = 0.26). Finally, mRNA-based testing significantly improved specificity, PPV and NPV of the DNA test (McNemar test, *p* < 0.001), while difference in sensitivities did not reach significance (Table 4).

## 3. Discussion

Biobanks, combining biological and medical methodologies, make possible a multidisciplinary approach to human health. Particularly, Biobanks comprised in clinical settings would represent the continuum by which to translate evidence-based medicine from the bench (research) to the bedside (clinical practice) [25]. For these reasons, Biobanks are on the list of the “10 Ideas Changing the World Right Now,” published in Time 2009 [26].

Modern medicine is regarded as “4P medicine”: personalized, preventive, predictive, and participatory. Biobanks would contribute to all of these characteristics. “Personalization” would reflect the achievements of tailored therapy basing on genome; “predictiveness” would predict the risk of diseases, by integrating genomic profile with age, sex, lifestyle, and environmental data; “prevention” would avoid or minimize risk factors for disease; “participation” would promote the active involvement of patients in the whole healthcare process [26].

Biobanks with large collection of archived specimens are particularly precious for epidemiological and long-term retrospective studies. [27,28,29]. Storage of tissues and cells with intact morphology, proteins, DNA and RNA, aiming research and diagnostic studies, should represent the first goal for a biobank [30,31,32]. Avoiding RNA degradation is a major challenge in this process, since this molecule has been demonstrated to be generally less stable than DNA with respect to various fixative [22,33,34,35]. Mostly, cross-linking between RNA and proteins would result in RNA-protein complexes, which reduce the efficiency of standard purification methods [36,37,38,39].

Ideally, tissues and cells should be immediately and completely fixed from the living state, since anoxia, changes in pH and other environmental factors may led to loss of RNA integrity. It has been shown that significant biochemical alterations occur in cells within 10 min after anoxia. Thus, the pre-fixation time should be kept at a minimum to minimize RNA degradation.

These issues can be prevented by freezing tissue and cells directly at −80 °C, by immersing them in liquid nitrogen or by placing them on ice until further processing [40]. These strategies would make the routinely collection of specimens more laborious and expensive.

Multiple studies indicate that ethanol and methanol are excellent fixatives for preserving nucleic acids degradation, since they bring about little chemical changes. Moreover, the low molecular weight and the rapid tissue penetration of the alcohol is thought to contribute to the uniform tissue and cells fixation with a minimal loss of tissue components [41].

PreservCyt is an alcohol-based fixative. In this medium, HPV-DNA was proved to be unaltered at RT for up to 9 years [32,36,37,38]. Turkowsky et al. found intact RNA for successful RT-PCR after one year of storage in PreservCyt [42]. However, their measurements were made on HPV-16 infected cell-line specimens, which might not reflect the characteristics of clinical samples.

Spectrophotometer 260 nm absorbance (A260) of 1.0 is equivalent to about 40 µg/mL of RNA. The A260/A280 ratio assesses RNA purity, by checking for possible proteins contamination. De facto, the presence of proteins, phenol or other contaminants strongly absorbing at or near 280 nm, would affect the subsequent use of purified RNA. A ratio of about 1.8 is generally accepted as “pure” for DNA; a ratio of 1.9–2.0 is generally accepted as “pure” for RNA. Lower ratios would indicate sample contamination [24,36,37].

For our analyses, total RNA has been isolated by using the fully automated EasyMag extraction platform that relies on cell lyses and magnetized silica dioxide particles. We obtained RNA of excellent yield and purity. As described by the literature, magnetic beads-based extraction methods provide a higher RNA yield and purity with less inhibitors in comparison with spin columns [43,44].

Total RNA obtained by long-term stored LBC samples demonstrated the same characteristics shown by the total RNA purified from samples stored at −20 and 4 °C. In addition, RNA purity from long-term LBC specimens did not significantly differ from those from snap-frozen tissues. The higher values for RNA concentration found in snap-frozen specimens would most likely reflect the higher cellularity of tissue specimens compared to cells counterparts. On the other hand, total RNA isolated from fresh-frozen tissue showed low yield and purity; this was probably due to the long pre-fixation time before freezing at −80 °C [35].

Spectrophotometer analysis provides a measure of the recovery and the purity of RNA but it is not suitable for the assessment of RNA integrity [45,46]. The integrity of purified RNA is crucial for RT-PCR-based techniques. We bridged the gap by using the human *U1A* housekeeping gene, enclosed in mRNA testing as endogenous internal control. Due to the low presence of U1A within human cells, a detectable level of this gene would confirm the integrity of the RNA molecule.

Our analyses demonstrated the low percentage (11.9%) of LBC samples in which U1A was not detected, as well as the weak correlation between the time of sample collection and the quality of RNA. This indicates that attention should be paid to the good preservation of this molecule in PreservCyt medium, stored at RT for a long time. Using U1A detection as the unique criterion to encode mRNA test as determined, previous studies demonstrated a percentage of invalid results ranging from 12.8 to 21% [47]. In these studies, specimens underwent mRNA testing within six weeks from collection (in accordance with manufacturer’s recommendations). Other studies, which considered as determined the tests in which HPV *E6/E7* mRNA was detected, even in the absence of U1A amplification, showed a rate of undetermined results ranging from 3 to 5% [48].

As showed by Figure 3, the profile of the U1A kinetic curve related to the sample collected in 2002 did not differ from those related to snap-frozen tissue, LBC samples stored at 4 °C and −20 °C respectively, and positive control included in the test. On the other hand, the shape of the kinetic curve related to the fresh-frozen specimen confirmed the low RNA content of this sample, at the same time assessing the excellent analytic sensitivity of the Nuclisens assay. These findings are particularly relevant, since mRNA testing is usually performed after cytological assessment and HPV-DNA test. In such cases, LBC specimens would stay at room temperature for several days before undergoing triage test.

Our study also investigated the suitability of mRNA testing in detecting clinically relevant disease. In accordance with recent studies, we considered CIN2+, instead of CIN3+, as the outcome threshold [7,49,50]. Based on a clinical setting in which colposcopy is used as triage method, as suggested by the European guidelines for quality assurance in cervical screening [51,52,53], we confirmed the higher level of specificity and PPV of mRNA testing with respect to DNA test. It is also known that the cross-reaction of HC2 with untargeted nononcogenic HPV types contributes to a reduction in the test specificity [54]. Thus, focusing on the detection of viral oncogenic activity, a reflex mRNA test was confirmed to be helpful for risk evaluation.

In a screening program, a triage test should demonstrate high clinical accuracy. Accuracy can be defined as the degree of conformity as compared with a gold standard that, for cervical pathology, is the histopathological diagnosis of CIN. Accuracy includes optimal diagnostic sensitivity and specificity, as well as a high detection rate of final endpoint disease.

In our casuistry, sensitivities for mRNA and DNA tests did not statistically differ, accordingly with Molden and coll. [54]. On the other hand, our findings are partially conflicting with Arbyn et al., [55] and Benevolo et al. [56], which demonstrated low sensitivity of Nuclisens assay, in comparison with HC2. In truth, Arbyn and coll., analysing Nuclisens as a triage test of minor cytological abnormalities, did not include any HSIL or SCC. In Benevolo and coll., SCCs were not comprised. Conversely, the inclusion of nine case of SCC in our casuistry (reaching 6% of the whole study population), strongly influenced the sensitivity of the mRNA test, since the five HPV types identified by this test account for 82 to 90% of all SCCs worldwide [57]. Stratifying the prevalence of mRNA positive results by cytological and histological grade, the sensitivity of the test improved with the severity of the lesion (data not shown).

Analysing results obtained by the application of the mRNA test on HPV-DNA-negative cases, we detected two CIN2+ cases resulting as mRNA positive. HC2 assay exclusively focuses on the detection of L1 structural region of HPV genome. Although the L1 region does play a role in the replication of the virus, it is not involved in the oncogenic mechanism [58]. In case of a loss of L1 region, due to viral integration into the human genome, DNA test will incorrectly define the sample as being negative, even though there could be aggressive activity of the virus, which could lead to cellular changes by *E6/E7* oncoproteins [59]. It has been estimated that up to 5% of cancers and CIN3 may not be detectable because of deletions of the HPV L1 region [59,60].

In our findings, women having a positive mRNA test result have a CIN2+ risk (OR) higher if compared with women with a positive DNA result. Since OR encloses both sensitivity and PPV, the higher value we found would indicate the overall better performances of mRNA test in identifying women who need further follow-up.

In our casuistry, the prevalence of high-grade lesions did not appear to be age-limited, although all of the invasive cancers occurred in the oldest patients. Considering the aim of determining whether it would be feasible to prioritize referrals to mRNA testing by age, giving priority to women aged 30 and older, we must conclude that it would not be necessary.

Finally, it is our opinion that the introduction of reflex mRNA test on behalf of cervical cancer algorithm is suitable to increase the accuracy of HPV-DNA testing, thereby reducing over-management and overtreatment. This tailored approach give hope to improve the effectiveness and appropriateness of cervical cancer prevention strategies.

## 4. Materials and Methods

### 4.1. Setting

A retrospective incidence-based study was performed by collecting data from the electronic Regional Cervical Cancer Registry linked to the regional tissues and cells Biobank, named Adriatic Biobank (AB). AB is placed in the Surgical Pathology Unit of the University Hospital of Chieti, Italy. Here, starting from 2001 until 2012, 43,000 residual cervical specimens collected from women participating in the Regional population-based cervical screening program were stored at room temperature (RT) and without any further handling. At the time of gynaecological examination, cervical specimens were collected from ecto-endocervix by Cervex Brush (Rovers Medical Devices, Oss, The Netherlands) and immediately immersed in 20 mL of PreservCyt solution (Cytyc Corporation, Boxborough, MA, USA).

A residual cervical specimen was the cellular material remaining in PreservCyt solution after the conclusion of the routine screening round.

The 43,000 residual cervical specimens stored in AB were related to 29,430 women. The characterization of the whole study population is shown in Figure 1. All patients were managed strictly, following the clinical indications related to their pathology and according to the National Guide Lines and Good Clinical Practice. Patients were referred for colposcopy because of (1) cytological evidence of LSIL or more severe; (2) persisting Atypical Squamous Cells of Undetermined Significance (ASCUS) at 6 months; (3) HPV-DNA positive result.

To assess the yield and the purity of the isolated RNAs, and to investigate the analytic performances of mRNA test, the following inclusion criteria have been applied: sample collection years ranging from 2001 to 2012; baseline ASCUS-or-more (ASCUS+) diagnosis; HPV-DNA test result; colposcopy-directed biopsy; availability of at least 4 ml of residual cytological specimen.

Diagnostic performances of *E6/E7* mRNA test was analysed on a subset of patients having a long-term follow-up (2 years, at least). Negativity for CIN2+ at the end of follow-up was defined by a negative cytology and a negative HPV-DNA test. In the event of ASCUS+ cytology or positive HPV-DNA test, CIN2+ negativity was determined by means of a negative colposcopy or by colposcopy-guided biopsy resulting as histologically negative or CIN1.

Exclusion criteria included: treatment for cervical lesion in the previous five years; history of any type of cancer; undergoing a surgical or ablative treatment, except biopsy, during baseline colposcopy: hysterectomy; pregnancy; HIV positivity or other causes of immunodeficiency.

For the purpose of the present study, final data were collected in May 2017.

### 4.2. Cytological and Histological Diagnosis

Cytological slides were prepared baseline by using the ThinPrep 2000 system (Cytyc Corporation, Boxborough, MA, USA), according to the manufacturer’s instruction. Slides were next stained using Papanicolaou method and evaluated according to 2001 Bethesda Reporting System as follows: ASCUS; Atypical Squamous Cells—cannot exclude High-grade SIL, ASC-H; Low-grade Squamous Intraepithelial lesion, LSIL; High-grade SIL, HSIL; Squamous Cell Carcinoma, SCC [61].

Hematoxylin-eosin tissue slides from colposcopic-guide biopsy were revised by two independent surgical pathologists, separately and blinded to all other study results. The final diagnosis was established based on the highest grade of Cervical Intraepithelial Neoplasia (CIN) detected, and in accordance with the 2005 World Health Organization (WHO) guidelines [62]. For the purpose of this study, benign cases (ectropion) and CIN grade 1 (CIN1) were referred here as less than CIN2 (CIN2−). CIN grade 2 (CIN2), CIN grade 3 (CIN3) and Invasive Squamous Cell Carcinoma (SCC) were referred here as Cervical Intraepithelial Lesions grade 2-or-worse (CIN2+). Only patients whose histological diagnosis reached consensus were finally included in the study. In our analysis, we assumed that both colposcopy and biopsy were 100% sensitive. In this view, histological diagnosis from colposcopy-guided biopsies were accepted as a verification of disease status, and was regarded as the gold standard [7]. Moreover, negative colposcopy was considered as sufficient ascertainment for absence of disease.

### 4.3. HPV-DNA Detection

HPV-DNA test was performed baseline to triage ASCUS+ cases using Hybrid Capture 2 technology (HC2, Qiagen, Gaithersburg, MD, USA) was used. The test relies on a signal-amplified hybridation platform for the semiquantitative detection of 13 most common high/risk HPV types (16, 18, 31, 33, 35, 39, 45, 51, 52, 56, 58, 59 and 68). HC2 reactions were read by a luminometer, which provided a relative quantification of each individual sample in comparison to the mean of a series of positive controls containing 1 pg/mL of HPV-DNA (corresponding to 100,000 HPV-16 genomes/mL or 5000 HPV copies per reaction). The cut-off of 1.0 relative light unit (RLU) was chosen to classify a specimen as positive or negative, in accordance with manufacturer’s instruction.

### 4.4. RNA Isolation

For the purpose of this study, an aliquot of each LBC residual specimen was retrospectively removed to perform an mRNA test. According to the manufacturer’s protocol, the volume of LBC sample to be used to perform an mRNA test ranges from 1 to 5 mL, depending on the nature of the specimen (number of cells). Given the high cellularity of cervical specimens collected in our setting, a volume of 4 mL was taken to match with the volume used to perform HPV-DNA test.

Each aliquot was transferred in a sterile 10 mL microtube and centrifuged at 2500 rpm for 12 min. Supernatant was removed and pellet underwent Nuclisens EasyMag system (Biomérieux, Marcy l’Etoile, France), an automated platform specifically optimized for total nucleic acids extraction from biological samples. This system automates an enhanced magnetic silica version of Boom technology. Nucleic acids were eluted from the solid phase in 55 μL of elution buffer and stored at −80 °C for pending analyses.

In order to compare the influence of transport media and storage under controlled conditions, RNA was also isolated from the following adjective samples: (i) one fresh-frozen tissue specimen, removed from a patient during hysterectomy. The lapse of time from surgical excision to refrigeration (at −80 °C) was of about twenty minutes; (ii) six snap-frozen specimens, removed from patients during hysterectomy. These samples were immediately placed on ice after excision and then stored at −80 °C [35]; (iii) one LBC specimen stored at 4 °C; (iii) one LBC sample stored at −20 °C.

Samples stored at 4 and −20 °C underwent the same purification protocol used for RT specimens.

Snap-frozen and fresh-frozen tissues were minced into small pieces (1–2 mm) with a sterile scalpel blade. Tissue was then transferred in a sterile 10 mL microtube where 1 mL of Lysis Buffer (Biomérieux) was added. The mix was then incubated at 95 °C for 15 min and centrifuged at 12,000 rpm for 5 min. Supernatant was removed and the pellet underwent automated nucleic acid extraction.

### 4.5. Quantification of RNA Yield and Purity

UV spectrophotometric absorbance (NanoDrop 2000 Technologie, Inc., Wilmington, DE, USA) was used to measure RNA yield (recovery) of each sample, and to determine its purity (ratio of sample absorbance at 260 and 280 nanometer wavelengths, 260/280 ratio). Measurements were performed on a single micro-litre of each RNA specimen [24].

### 4.6. E6/E7 mRNA Test

A total of 15 μL of nucleic acid from each RNA sample was used to retrospectively perform *E6/E7* mRNA testing (henceforth mRNA test), by Nuclisens EasyQ HPV v1 test (Biomérieux, Marcy l’Etoile, France), in accordance with manufacturer’s instructions. Nuclisens EasyQ is based on real-time nucleic acid sequence based amplification (NASBA) procedure, which utilizes molecular beacon probes labelled with 5-carboxyfluorescein (FAM) and Texas Red fluorochromes, at an isothermal temperature of 41 °C. Molecular beacons are single-stranded oligonucleotide probes with a stem-loop structure that fluoresce only upon hybridization with the target. One arm of the stem is labelled with the fluorescent dye and the other with a nonfluorescent quencher. In the nonhybridized state, the fluorescent signal is captured by the quencher and released as heat; upon hybridization, the fluorescent dye and the quencher are separated and a fluorescent signal is transmitted.

The test identifies full-length *E6/E7* mRNA from five high-risk carcinogenic HPV types (16, 18, 31, 33 and 45) by a “real-time” Reverse Transcription-Polymerase Chain Reaction (RT-PCR). RNA amplification and relative accumulation of target-specific fluorescent signal occur simultaneously. A fluorometer (Nuclisens EasyQ analyser) detects fluorescence and generates plots over time. mRNA testing was defined as positive by Nuclisens analysis software, if at least one of the five HPV genotypes detected by the test has been found. The human *U1A* gene is used as endogenous internal control to assess both RNA integrity/adequacy and accuracy of the amplification processes. U1A is a low abundance house keeping gene, encoding for a small ribonucleoprotein protein. It is provided in one freeze-dried accusphere that will be diluted in the corresponding buffer and used directly for amplification. One specific molecular beacon is used for U1A amplicons. For our purpose, when U1A mRNA was not detected even if in presence of *E6/E7* mRNA from one of the five HPV types, the test was encoded as “undetermined”.

To evaluate the run validity, positive controls for HPV16, 18, 31, 33 and 45 as well as negative control (RNA-free water) were added in each run.

Presently, Biomérieux ensures PreservCyt cervical samples as suitable to perform mRNA testing for up-to six weeks, at room temperature [47]. In order to provide comparative data, we included in the study 202 consecutive cervical samples undergoing mRNA test within six weeks from collection.

### 4.7. Statistical Analyses

Pearson’s index and chi square test were applied to investigate the correlation between the year of LBCs collection and the integrity of RNA (amplification of the *U1A* gene).

By standard methods, the authors calculated the prevalence of positive results for both HPV-DNA and *E6/E7* mRNA tests. 2 × 2 tables were used to associate variables and frequencies. The clinical performances of molecular tests were calculated with 95% confidence intervals (CIs) based on histological diagnosis. McNemar test was applied to evaluate statistical significance. Odds ratio (OR) was used to assess the association between molecular tests and hitological outcome.

By chi square test (or Fisher’s exact test, when appropriate) we tested the differences between variables. Concordance between HPV-DNA test and HPV-mRNA test was assessed by Kappa statistics. According to the criteria of Landis and Koch, the K values were divided into six scales of strength of agreement: poor (<0.00), slight (0.00–0.20), fair (0.21–0.40), moderate (0.41–0.60), substantial (0.61–0.80) or almost perfect (0.81–1.00) [63].

To evaluate the trend of mRNA tests results in relation with the severity of cervical lesion, Cochran-Armitage test was used. Statistical tests were performed by using SPSS software (SPSS for windows, Inc., Chicago, IL, USA), version 15.0. In all analyses, probability values *p* less than 0.05 were regarded as significant.

### 4.8. Ethical Aspects

The study, approved by G. d’Annunzio University of Chieti-Pescara and by Local-Health-Authority, was performed by respecting the prevailing norms of Good Clinical Practice and in accordance with the principles outlined in the Declaration of Helsinki (1975, 2008 revision).

At the time of gynaecologic examination, each woman gave a written informed consent to provide a residual specimen for scientific research. Sample identification code was assigned to each specimen entering in Biobank collection, in accordance with confidentiality standards.

## 5. Conclusions

The application of molecular techniques in clinical settings is still evolving.

Our findings would emphasize that: (i) exfoliated cervical cells are stable in PreservCyt medium for extended periods, even when stored at RT; (ii) in RT LBC samples, RNA is well preserved and can effectively be isolated using automated extraction procedures; (iii) the time of RT storage does not significantly adversely affect the performance of the *E6/E7* mRNA assay.

Due to the increasing use of molecular testing in clinical settings, our observations would answer to many authors who required data concerning the effects of long-term RT storage on tests’ performances [64,65,66,67]. PreservCyt samples, even if stored at RT, provide good RNA. Moreover, room temperature storage would imply lower spatial, fiscal and environmental costs, if compared with cold storage and processing [67,68].

We believe that a “one sample for all” approach is possible.

The use of a medium that is routinely used in clinical settings would undoubtedly be realistic and have practical benefits for healthcare services. [41,69,70].

## Figures and Tables

**Table 1:** Distribution of results from mRNA testing, in accordance with clustered years of collection.

Years of Sample Collection	*E6/E7* mRNA Test Result (%)		
	Determined °	Undetermined *	Total (%)
2001–2004	52 (29.2)	4 (16.7)	56 (27.7)
2005–2008	81 (45.5)	14 (58.3)	95 (47)
2009–2012	45 (25.3)	6 (25)	51 (25.3)
Total (%)	178 (88,1)	24 (11.9)	202

° *U1A* gene was detected, * *U1A* gene was not detected.

**Table 2:** Distribution of study population according to histological diagnosis (dichotomised as Cervical Intraepithelial Neoplasia grade 2, (CIN2−, and CIN2+), cytological diagnosis, human Papillomavirus (HPV)-DNA and *E6/E7* mRNA testing.

Hystological Diagnosis (%)	Cytological Diagnosis (%)				HPV-DNA Test (%)		*E6/E7* mRNA Test (%)		
	ASCUS	LSIL	ASC-H/HSIL	SCC	Positive	Negative	Positive	Negative	Undetermined *
CIN2−	24 (54.5)	23 (43.4)	9 (20.9)	0	44 (32.6)	12 (85.7)	10 (10.8)	31 (97)	15 (62.5)
CIN2+	20 (45.5)	30 (56.6)	34 (79.1)	9 (100)	91 (67.4)	2 (14.3)	83 (89.2)	1 (3)	9 (37.5)
Total	44 (29.5)	53 (35.6)	43 (28.9)	9 (6)	135 (90.6)	14 (9.4)	93 (62.4)	32 (21.5)	24 (11.9)

CIN2−, Cervical Intraepithelial Neoplasia less than grade 2; CIN2+, Cervical Intraepithelial Neoplasia grade 2-or-worse; ASCUS, Atypical Squamous Cells of Undetermined Significance; LSIL, Low-grade Squamous Intraepithelial Lesion; ASC-H, Atypical Squamous Cells-cannot exclude High-grade SIL; HSIL, High-grade Squamous Intraepithelial Lesion; SCC, invasive Squamous Cell Carcinoma. * *U1A* gene was not detected.

**Table 3:** Clinical performances of *E6/E7* mRNA test in relation with age of patients.

Age, Years	Clinical Performances of *E6/E7* mRNA Test, % (95% CI)			
	Sensitivity	Specificity	PPV	NPV
<30 (*n* = 38)	96.3 (81.7–99.3)	63.6 (35.3–84.8)	86.7 (74.5–98.8)	87.5 (64.6–100)
≥30 (*n* = 87)	100 (92.3–100)	80 (62.2–90.7)	90.5 (83.2–97.7)	100 (100–100)

CI, Confidence Intervals; PPV, Positive Predictive Value; NPV, Negative Predictive Value.

**Table 4:** Comparison of clinical performances of DNA and mRNA testing, for CIN2+ outcome.

Molecular Testing	Clinical Performances, % (95% CI)			
	Sensitivity	Specificity	PPV	NPV
HPV-DNA	97.6 (90.8–99.5)	17 (7–32.6)	70.7 (61.4–78.6)	77.8 (40.2–96)
HPV *E6/E7* mRNA	98.8 (92.6–100)	75.6 (59.4–87)	89.4 (80.7–94.4)	97 (82–100)

CI, Confidence Intervals; PPV, Positive Predictive Value; NPV, Negative Predictive Value.
